# The Interplay of Inflammatory Bowel Disease (IBD) and Diabetes in Pediatrics: A Systematic Review

**DOI:** 10.7759/cureus.70425

**Published:** 2024-09-29

**Authors:** Amir Fayyaz Shaikh Sardar Muhammad, Talal Mohammad K Abdulkareem, Abdullah Enayatullah Bakheet Alharbi, Nujud Abdullah Alessa, Salwa Bin Qaed, Ebrahim Khalil Ebrahim, Elaf J Zurayyir, Muna Alnory S Alqasem, Jazza Aamir

**Affiliations:** 1 Pediatrics, Maternity and Children Hospital, Arar, SAU; 2 Medicine and Surgery, King Abdulaziz Medical City, Riyadh, SAU; 3 General Practice, King Salman Hospital, Riyadh, SAU; 4 Medicine and Surgery, Armed Forces Hospital, Dhahran, SAU; 5 Medicine, King Khalid University, Abha, SAU; 6 Pediatric Emergency Medicine, Qatif Central Hospital, Eastern Health Province, Qatif, SAU; 7 Medicine, Jazan University, Jazan, SAU; 8 Medicine and Surgery, National Guard Health Affairs, Riyadh, SAU; 9 Medicine and Surgery, Dow University of Health Sciences, Karachi, PAK

**Keywords:** children, crohn’s disease, diabetes mellitus, inflammatory bowel disease, systematic review, ulcerative colitis

## Abstract

The main objective of this study is to provide insights into the clinical problems and considerations in managing pediatric patients with both diabetes and inflammatory bowel disease (IBD) by summarizing the available information. We conducted a comprehensive search across electronic resources, including ScienceDirect, PubMed, Cochrane Library, and Scopus. Two independent reviewers evaluated and retrieved information from qualifying papers. Our data consists of five studies with 79,878 patients, 38225 (47.9%) of whom were female. Three studies included 2432 children diagnosed with IBD, and 17 (0.7%) were found with type 1 diabetes (T1D). Two studies comprised 77,446 children diagnosed with T1D and 83 (0.1%) had IBD. Children with immune-mediated diseases are more likely to have IBD, especially Crohn's disease. The included studies found no connection between T1D and childhood IBD. The important but little-known connection between diabetes and IBD in pediatric populations is brought to light by this comprehensive study. We were unable to discover a connection between pediatric IBD and DM. The review does, however, point out significant gaps in the literature, highlighting the need for more studies to comprehend the intricate interactions between these disorders and to create practical management plans for impacted children.

## Introduction and background

While the precise etiology of inflammatory bowel disease (IBD) remains unclear, the fact that host-related, environmental, and hereditary factors contribute to the development of inflammation and intestinal fibrosis is well-acknowledged [1]. According to recent research, people with IBD have an increased chance of getting multiple sclerosis and psoriasis, among other autoimmune disorders [2,3]. Due to rising obesity rates, declining levels of physical exercise, and aging populations, Worldwide, there has been a notable surge in the prevalence of diabetes mellitus (DM). Based on certain projections, the prevalence is expected to rise quickly from 2.8% in 2000 to 4.4%-7.7% in 2030 and up to 9.9% of the entire population in 2045 [4,5]. Furthermore, the development of DM is influenced by a combination of environmental and genetic variables, such as infections, nutrition, gut microbiota, and variations among the genes for PTPN2, IFIH1, INS, and HLA [6,7].

It has been proposed that there may be an epidemiological connection between type 1 diabetes (T1D) and IBD due to their shared immune-mediated pathophysiology [8]. A new meta-analysis found no link between IBD and T1D. Subgroup research, however, suggests that individuals without IBD may have a lower risk of developing type 1 diabetes than people with Crohn's disease (CD) or ulcerative colitis (UC) [9].

Though it may affect the choice of medication and related results, the impact of coexisting type 1 and type 2 diabetes on the onset of IBD has not been extensively researched [10]. More importantly, diabetes seems to increase the chance of illness and mortality from all causes [11], as evidenced by several recent studies that link the condition to higher severity [12].

IBD and DM are two chronic diseases that have major long-term health effects. More people are becoming aware of their coexistence, especially in young populations. These disorders are becoming more common in children, which raises questions about how they can interact and what that means for patient outcomes and illness management. Although there is ample evidence linking IBD and diabetes in adults, the relationship in children is still little understood, even though there may be particular pathophysiological linkages at play as well as effects on development, growth, and quality of life [12]. This study aims to review studies on the relationship between diabetes and IBD in pediatric populations.

## Review

As per the guidelines provided by the Preferred Reporting Items for Systematic Reviews and Meta-Analyses (PRISMA), a methodical evaluation was carried out [13]. SCOPUS, PubMed, Web of Science, Cochrane Library, and ScienceDirect were the electronic databases searched, among other bibliographic databases. English-language research on the application of the relationship between IBD and DM in children was the focus of our search approach. To guarantee a comprehensive search, we employed pertinent terms associated with both IBD and DM. In order to preserve neutrality, two impartial reviewers went through the search results, chose studies that fit the inclusion requirements, took out data, and used reputable assessment instruments to rate the included research's methodological quality.

Eligibility criteria

The inclusion criteria for the studies examined in this review are as follows: only research that explores the relationship between IBD and DM in children was considered. The focus is specifically on studies involving individuals under 18 years of age and those published in English. Eligible study designs include randomized controlled trials, observational studies, and cohort studies, whether retrospective or prospective, as well as cross-sectional and case-control studies. In contrast, the exclusion criteria specify that studies unrelated to the connection between pediatric IBD and DM were disregarded. Additionally, studies published in languages other than English, along with case studies, opinions, letters, reviews that lack original research, and conference abstracts will also be excluded from the review.

Data extraction

All search results were reviewed for screening, including titles and abstracts accuracy and consistency by using pre-established inclusion and exclusion criteria to determine their relevance to the research issue. To promote effective screening and lessen bias, reference management software such as Rayyan (QCRI) was used [14]. Research that at least one reviewer thought to be pertinent was advanced to full-text inspection by both reviewers. All disputes pertaining to inclusion were settled by consensus and dialogue. Important information was obtained from the research included using a standardized information extraction form, including titles, authors, publication year, research setting, participant demographics (age and gender distribution), population type, prevalence of IBD/ DM according to the type of population, and primary outcomes. In addition, an established tool for methodological quality assessment was used to evaluate the risk of bias in the included research.

Data synthesis methodology

Using information from relevant studies, summary tables were made to provide a qualitative overview of the research findings and elements. The best manner to use the data from the research included in the review will be determined when the systematic review's data collection is finished.

Peril of prejudicial evaluation

The critical evaluation standards for studies reporting incidence data from the Joanna Briggs Institute (JBI) were used to evaluate the study's quality [15]. The tool has nine questions. A score of one is assigned to affirmative answers, while a score of zero is given to negative, unclear, or irrelevant answers. Less than four, five to seven, and eight or more will be classified as low, moderate, and high quality, respectively. Each researcher evaluated the study's quality independently, and disagreements were settled through discussion.

Results

Search Outcomes

**Figure 1:**
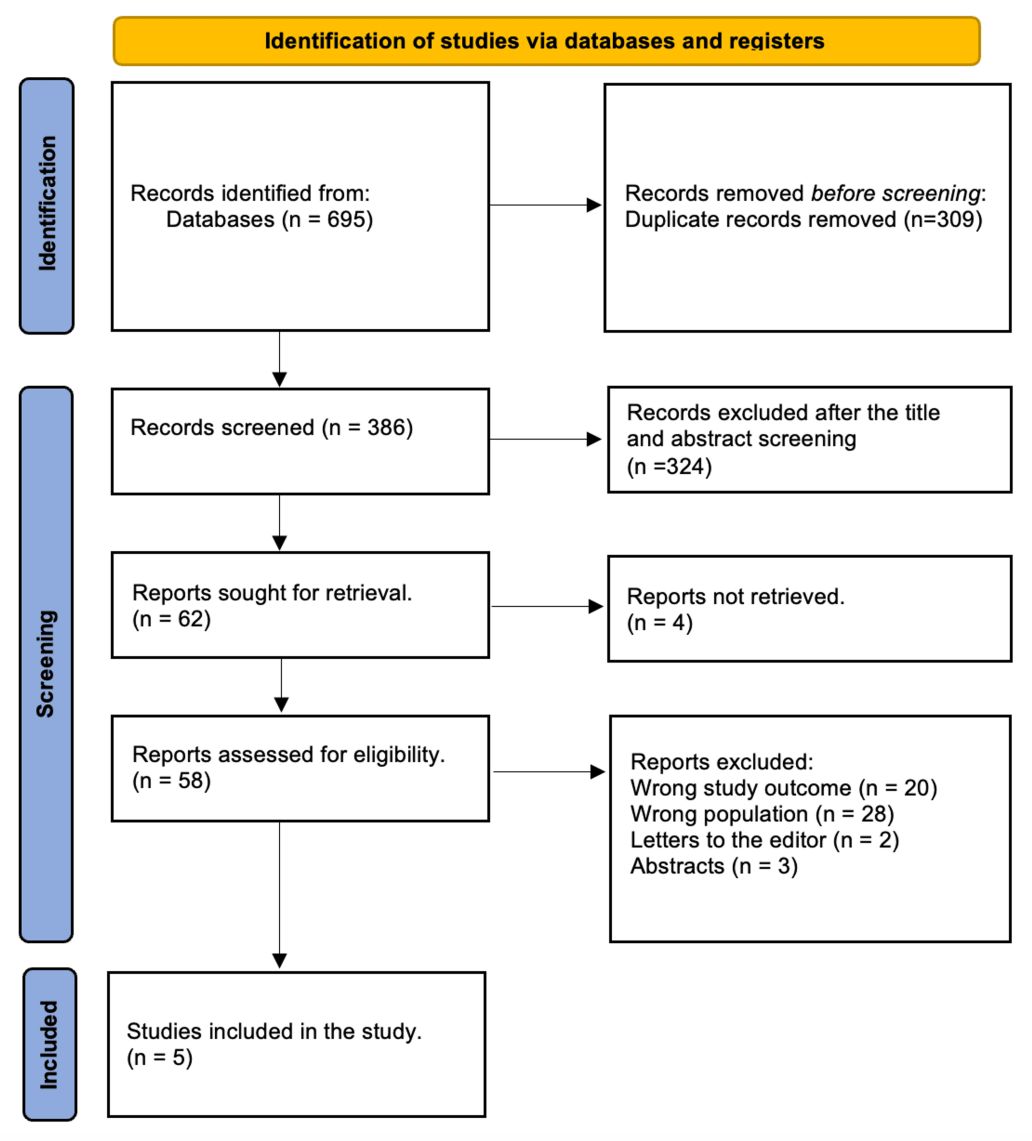
Study's decision-making is summarized in a PRISMA diagram. PRISMA: Preferred Reporting Items for Systematic Reviews and Meta-Analyses

Following the removal of 309 duplicates, a thorough search yielded 695 study publications. After 386 studies' titles and abstracts were reviewed, 325 papers were rejected. Four of the 62 reports that needed to be retrieved could not be found. After 58 articles were screened for full-text review, 20 were discarded due to inaccurate research findings, and 28 were rejected due to inappropriate population type, 3 were abstracts, and 2 were editor's letters. This systematic review included five research papers that satisfied the qualifying requirements. There were five research publications in this systematic review that met the eligibility criterion (Figure 1).

Sociodemographic Parameters of the Researched Subjects

Table 1 illustrates the demographic data from the research articles. Our data consists of five studies with 79,878 patients, 38225 (47.9%) of whom were female [16-20]. Four studies were retrospective cohorts [17-20] and one was a cross-sectional study [16]. The research was done as recently as 2011 [16] and as recently as 2024 [19].

**Table 1:** Factors related to the targeted populations' sociodemographics.

Study	Study design	Country	Participants	Mean age	Females (%)
Kappelman et al., 2011 [16]	Cross-sectional	USA	1242	15 ± 3.4	559 (45%)
Virta et al., 2013 [17]	Retrospective cohort	Finland	595	10.2	256 (43%)
Ghersin et al., 2020 [18]	Retrospective cohort	Israel	595	17.1	293 (49.2%)
Samuelsson et al., 2024 [19]	Retrospective cohort	Sweden	12,272	9.5 ± 4.4	6774 (55.2%)
Jasser‐Nitsche et al., 2021 [20]	Retrospective cohort	Austria	65,174	14 ± 3.9	30,343 (46.6%)

Clinical Results

The parameters related to the clinical state are shown in Table 2. Three studies included 2432 children diagnosed with IBD, and 17 (0.7%) were found with T1D. Two studies comprised 77,446 children diagnosed with T1D and 83 (0.1%) had IBD. Children with immune-mediated diseases are more likely to have IBD, especially CD [16,20]. The included studies found no connection between T1D and childhood IBD [17-19].

**Table 2:** Clinical aspects and outcomes of the studies discussed in this review. IBD: Inflammatory bowel disease; T1D: type 1 diabetes; JBI: Joanna Briggs Institute

Study ID	Operation	Prevalence of IBD/T1D (%)	Main outcomes	JBI
Kappelman et al., 2011 [16]	Children with IBD	15 (1.2%)	Children with immune-mediated diseases are more likely to have IBD, especially CD.	Moderate
Virta et al., 2013 [17]	Children with IBD	2 (0.3%)	They found no connection between T1D and childhood IBD.	High
Ghersin et al., 2020 [18]	Children with IBD	0	They found no connection between T1D and childhood IBD.	Moderate
Samuelsson et al., 2024 [19]	Children with T1D	20 (0.2%)	They found no connection between T1D and childhood IBD.	Moderate
Jasser‐Nitsche et al., 2021 [20]	Children with T1D	63 (0.09%)	When individuals with T1D experience gastrointestinal issues, they advise screening for IBD, despite the exceedingly low frequency.	Moderate

Discussion

According to our knowledge, this is the first systematic review to investigate the relationship between IBD and DM among children. Auto-immune mediated and chronic diseases are reported to be associated. However, we included a specific population (i.e. children). We found a lack of literature and with a small sample size in three out of the five studies. Three studies included 2432 children diagnosed with IBD, and 17 (0.7%) were found with T1D. Two studies comprised 77,446 children diagnosed with T1D and 83 (0.1%) had IBD. Children with immune-mediated diseases are more likely to have IBD, especially CD [16,20]. The included studies found no connection between T1D and childhood IBD [17-19]. In line with our results but in the adult population, Lu et al. found that there was no epidemiologic correlation between T1D and IBD, according to the entire meta-analysis [9]. Subgroup analysis results stratified by the study region, however, suggested that individuals with IBD from particular regions would be more likely to acquire T1D [21].

When combined, T1D and IBD can have a similar pathophysiology. It is possible that individuals with IBD have a higher risk of T1D. Epidemiological research, however, has shown mixed findings. Previously, genetic data revealed a substantial association between T1D and IBD. IBD and T1D shared risk polymorphisms at 20 loci, which is 10 times greater than that predicted by chance, according to a genome-wide association study [22].

Furthermore, compared to healthy controls, clinical research has shown that patients with both T1D and IBD have a different gut microbiota composition and less diversity [23,24]. The growth and operation of the human immune system are significantly influenced by microorganisms, and disruptions in gut microbiota can lead to a dysregulated inflammatory response. For example, the gut microorganisms release short-chain fatty acids, which have significant anti-inflammatory properties [25].

Ananthakrishnan et al. reported that DM was a distinct risk factor for infections in IBD patients undergoing immunomodulatory medication, including pneumonia, UTIs, and sepsis [25]. Kumar et al. discovered that having DM together with IBD increased the incidence of sepsis, pneumonia, UTIs, and skin and soft tissue infections [12]. Choi et al. identified DM as a covariate linked with an increased incidence of CD-related hospitalizations [26]. Din et al. found that patients with DM and IBD had higher healthcare utilization than patients with IBD alone [11]. In terms of IBD treatment, DM patients appeared to utilize fewer biologics and immunomodulators. In contrast, we discovered increasing use of 5-ASA drugs, which are typically utilized in patients with lesser illness. This could be attributed to professionals' concern about infection in diabetic patients. However, it is possible that patients with DM have less severe IBD and hence require fewer advanced medications. A meta-analysis published in 2017 showed that no treatment option has a higher risk of severe infection than another, while broad confidence intervals indicated that a clinically relevant difference could not be excluded [27]. More research in this area is needed to determine whether DM, the type of DM, and DM therapy can influence the course of IBD and its medical and surgical treatments.

Limitations

It is important to recognize the various limitations of this systematic review. This is the first systematic review to investigate the relationship between T1D and IBD among a special population such as pediatrics. Thus, we barely found similar literature to compare and interpret our results. Numerous research studies used tiny sample sizes, which might have limited how broadly the results can be applied. Furthermore, there is a chance of publication bias because research with null or negative outcomes might not get the attention it deserves. The lack of longitudinal studies that monitor the long-term outcomes of kids with diabetes and IBD is another drawback. These studies would offer more thorough insights into the genesis and evolution of these illnesses.

Future studies ought to concentrate on filling in the gaps this review found. For the purpose of better understanding the prevalence and effects of diabetes in pediatric IBD populations, larger, multicenter research using standardized approaches is required. When evaluating the long-term effects and possible problems of various illnesses coexisting, longitudinal studies are especially crucial. Research is also required to establish integrated care models and treatment plans that cater to the special requirements of young patients with diabetes and IBD. Examining the immunological and genetic processes that underlie the connection between these illnesses may also yield important information for focused treatment approaches.

## Conclusions

The important but little-known connection between diabetes and IBD in pediatric populations is brought to light by this comprehensive study. There was no correlation between DM and IBD in children that we could discover. The review does, however, also point out significant gaps in the literature, highlighting the need for more studies to comprehend the intricate interactions between these disorders and to create practical management plans for impacted children.
